# Associations of lack of voluntary private insurance and out-of-pocket expenditures with health inequalities. Evidence from an international longitudinal survey in countries with universal health coverage

**DOI:** 10.1371/journal.pone.0204666

**Published:** 2018-10-09

**Authors:** Stéphanie Baggio, Marc Dupuis, Hans Wolff, Patrick Bodenmann

**Affiliations:** 1 Division of Prison Health, Geneva University Hospitals and University of Geneva, Geneva, Switzerland; 2 Life Course and Social Inequality Research Centre, University of Lausanne, Lausanne, Switzerland; 3 Institute of Psychology, University of Lausanne, Lausanne, Switzerland; 4 Vulnerable Population Center, Department of Ambulatory Care and Community Medicine, University of Lausanne & Lausanne University Hospital, Lausanne, Switzerland; West Chester University of Pennsylvania, UNITED STATES

## Abstract

**Objectives:**

In countries with universal health coverage (UHC), national public health insurances cover 70% of health expenditures on average, but health care user fees and out-of-pocket expenditures have been neglected in empirical patient-centered health inequality research. This study is the first to investigate how health care-related factors are associated with health status among middle-aged and elderly people—vulnerable groups for the burden of illness—in countries with UHC.

**Design:**

Longitudinal observational cohort study.

**Setting:**

Population-based cohort Survey of Health, Ageing and Retirement in Europe (SHARE) in twelve countries with UHC.

**Participants:**

Non-institutionalized people aged 50 and older (n = 29,260). Two subsamples were also used: participants without global activity limitation at baseline (n = 16,879) and participants without depression at baseline (n = 21,178).

**Main outcome measures:**

Risk of death, risk of global activity limitations, and risk of depression. We used mixed-effects Cox proportional hazards regressions to estimate hazard ratios (HR) for all-cause mortality, physical limitations, and depression.

**Results:**

Having a voluntary private insurance to cover health expenses not included in the public health care system (44.1% of the total sample) was a protective factor for all outcomes (HR≤0.91), controlling for a large range of socio-economic variables. On the contrary, having out-of-pocket expenditures (62.4%) was a risk factor (HR≥1.12).

**Conclusions:**

UHC systems are not free from health inequalities: there is a potential effect of lack of voluntary private insurance and out-of-pocket expenditures on mortality and health. Health care-related factors should be at focus in future researches designed to understand and address health inequalities. Reducing out-of-pocket expenditures and developing voluntary private insurance may protect against premature illness and death.

## Introduction

Universal health coverage (UHC) is a major health and political concern worldwide. It has been described as the best way to achieve health equity [1]. Health inequalities can be defined as differences in health status (e.g., mortality, mobility, body mass index) between individuals and groups (e.g., racial/ethnic disparities, gender disparities, income) [2]. One of most important health inequality is the inequitable access to health care [3]. During the past three years, several countries have successfully switched to a publicly financed health system [4]. However, even in countries with UHC, health inequalities remain [4, 5]. Indeed, public health insurance is always partial [6], even if it lowers the risk of catastrophic health care expenses and improve the access to health care [7]. For example, in most countries of the Organization for Economic Co-operation and Development, national public health insurances cover an average of 70% of health expenditures.

Wealth inequalities and other social determinants are well-known causes of health inequalities [8, 9], but health care-related factors may also be a cause of these health inequalities. A recent editorial suggested that health care user fees should be abolished because they create health care inequalities [1] and out-of-pocket payments are associated with an unmet need for health care [10]. However, few empirical studies investigated these potential factors of health inequalities. A Spanish study reported that women with voluntary private health insurance were more likely to use preventive health services than women who did not [11]. Associations of out-of-pocket expenditures with health inequalities have been studied in middle and low-income countries, where out-of-pocket expenditures correspond to a decrease in health care use [12]. Overall, research regarding the association between health care related factors and health inequalities in countries with UHC remains scarce. Such investigations are needed to highlight the potential effect of health care users fees on health inequalities.

Health inequalities persist in middle- and old-age with the accumulation of lifelong socioeconomic difficulties and vulnerability (i.e., the fragility of people’s lives and the incapacity to protect their own interests) [13] and the greater need for health care compared with other stages of life. Middle-aged and elderly people are one of the most vulnerable groups for health care burden [10], but they have been neglected in empirical patient-centered health inequality research [14]. In an ageing world with a rising proportion of elderly people, this question is particularly salient [15].

Thus, this study aimed to investigate how health inequalities among middle-aged and elderly people persisted in countries with UHC. The study assessed the potential effect of the lack of voluntary private insurance and out-of-pocket expenditures on mortality and indicators of physical and mental health (physical limitations and depression) of middle-aged and elderly people in countries with UHC.

## Materials and methods

### Study design

This study is a sub-analysis of data collected in the population-based cohort Survey of Health, Ageing and Retirement in Europe (SHARE) [16–18]. It focuses on non-institutionalized people aged 50 and older. The data analyzed include eleven European countries (Austria, Belgium, Denmark, Germany, Greece, Italy, France, Netherland, Spain, Sweden, and Switzerland), plus Israel. These countries were covered from the first wave in 2004 and in the following five waves until 2015, except Greece, which was covered in the three first waves (ending in 2009) and in the sixth wave (2015). A probability sample was used in countries in which registers of individuals were available (in most countries, there were stratified by age). In three countries in which no register was available (Austria, Greece, and Switzerland), pre-screening in the field were used to identify eligible sample participants. Participants were eligible for the study inclusion of they were born in 1954 or earlier, spoke the official language of the country, did not live abroad or in an institution (prison or institutions for elderly). Data were collected in 2004 using a computer-assisted personal interview program with an additional self-completed pencil and paper questionnaire for sensitive questions. The SHARE study was reviewed and approved by the Ethics Committee of the University of Mannheim (waves 1 to 4) and the Ethics Council of the Max Planck Society (waves 4 to 6).

A total of 31,161 participants were included at baseline in 2004. The average response rate was 61.6%, ranging from 38.8% in Switzerland to 81.0% in France. The attrition rate from the beginning of the study until the end was quite low (6.5% of the baseline sample). Some participants were excluded in subsequent waves (in total 54.4% of the baseline sample) because: 1) their household was not part of the wave, 2) they were not listed as members of households, or 3) they were dead. In the sixth wave in 2015, 11,446 participants were included in the study (among participants included in the first wave). For analyses on mortality, we used the whole sample. For analyses on physical limitations, we included participants without physical health problems at baseline (n = 17,918). For analyses on depression, we included participants without depression at baseline (n = 22,153).

### Measures

Health status was assessed using three outcomes: mortality, physical limitations, and depression.

#### Mortality

Participants’ ages and years of death (all-cause mortality) were recorded from the beginning of the study and until the sixth wave.

#### Physical limitations

We used the Global Activity Limitation Indicator (GALI), to assess long-term health-related disability (binary outcome coded “not limited” versus “limited”). This measure underlies the European indicator Healthy Life Years and is widely used to compare population health across different countries [19].

#### Depression

Mental health was measured using EURO-D, a 12-item self-reported questionnaire for depression (cut-off score ≥ 4) [20].

Physical limitations and depression were completed at baseline (2004), in the second wave (2006), in the fourth wave (2008), in the fifth wave (2013), and in the sixth wave (2015).

Risk factors for health inequalities were assessed at baseline and included demographics and socioeconomic status, health behaviors, and health care factors.

#### Health care-related factors

We measured two individual-level factors related to health care: having or not a voluntary private health insurance and out-of-pocket expenditures during the previous twelve months (recoded as: 0€, 1 to 199€, and 200€ or more –200€ being the median for participants having out-of-pocket expenditures). It included hospital (inpatient and outpatient) care, all kinds of consultations with health professionals (including dentists, exams, and therapies but not alternative medicines), drugs (excluding self-medication and drugs not prescribed), and day car/nursing care/home-based care.

#### Demographics and socioeconomic status

These individual-level variables included age, gender, marital status (single, divorced, or widowed versus being in couple or married), and income (using four categories corresponding to quartiles).

#### Health behaviors

Lifetime smoking (ever smoked daily, “yes/no”) and sport activity (“never or hardly ever” versus “yes”) were assessed. Participants were also asked whether they had or not forgone care because of financial problems during the previous twelve months for all kind of health care: surgery, care from a general practitioner, care from a specialist physician, drugs, dental care, hospital (inpatient) rehabilitation, ambulatory (outpatient) rehabilitation, aids and appliances, care in a nursing home, home care, paid home care, and any other care.

#### Type of UHC

At the system-level, the type of UHC was recorded for each country. There were three different models: 1) insurance mandate: The government mandates all citizens to purchase insurance; 2) two-tier: The government mandates minimum insurance coverage and allows purchasing additional voluntary insurance; and 3) single-payer: The government provides insurance for all residents and pays for all expenses.

#### Type of voluntary private insurance

At the system-level there are also differences between different types of voluntary private insurance. We can distinguish between three main types: duplicate, supplementary, and complementary coverage [21–23]. The duplicate system covers services already included in the mandatory health insurance. It increases users’ choice and improve access to health services. The supplementary system provides a coverage for health services not included in the mandatory health insurance. Finally, the complementary system provides a coverage for health services that are not totally covered by the mandatory health insurance and covers the residual costs. Some countries have a combination of these different types, as shown in Table 1.

**Table 1:** Baseline characteristics of countries (system level).

	Type of health insurance	Type of voluntary private insurance	% private insurance	% out-of-pocket expenditures	% forgone care	Response rate (%)
Austria	IM	C + S	25.1	71.1	2.6	55.6
Belgium	IM	S	75.3	58.2	3.3	39.2
Denmark	TT	C + S	36.4	77.0	1.5	63.2
France	TT	C	80.7	23.9	6.3	81.0
Germany	IM	C + S	13.8	74.6	5.5	63.4
Greece	IM	D + S	91.3	74.3	6.3	63.1
Israel	TT	D	48.0	77.5	14.1	60.1
Italy	SP	C + D	5.6	70.0	5.1	54.5
Netherland	TT	S	66.8	44.2	2.1	61.6
Spain	SP	D + S	9.6	34.3	2.9	53.0
Sweden	SP	C + S	9.3	85.6	2.8	46.9
Switzerland	IM	S	33.8	80.4	3.7	38.8

IM: insurance mandate, TT: two-tier, SP: single-payer, C: complementary, D: duplicate, S: supplementary.

Baseline data: 2004.

### Statistical analyses

First, descriptive statistics for individual- and system-level factors were computed. We then used mixed-effects Cox proportional hazards models to assess the relationship between the three health outcomes and individual and system-level factors. The country was used as a second-order factor in these multilevel models and to take into account that participants are nested in countries and the corresponding unobserved heterogeneity. Our study did not aim to compare countries, which would be difficult because of the important differences in public and private institutional variations in health insurance. Three separate models were developed for 1) mortality, 2) physical limitation, and 3) depression. The time variables used in these models were survival time, survival time without physical limitations, and survival time without depression, respectively, from the date of enrollment (2004). Participants who respectively survived and remained physically and mentally healthy were censored at the end of the study period (2015) or when they left the survey. Individual- and system-level covariates were included as predictors in the models. Individual covariates included health care-related factors, demographics, socioeconomic factors, and health behaviors. Since physical limitations and depression were not measured as continuous scales, we used the Efron method to handle tied events. This approximation has been demonstrated to perform as well as discrete models, which are not available for mixed survival models [24]. Countries were considered a second order level with random intercepts. Hazard ratios (HR) were reported. We used a backward selection model in order to have parsimonious models. Missing values were list wise excluded for the three models, except for financial-related variables (income and out-of-pocket expenditures), which had more than 5% of missing values. For these two variables, an additional category for participants who did not answer was created. It yielded a total of n = 27,515 (94.0% of the sample) in mortality model, n = 16,022 (94.9% of the subsample) in physical limitations model, and n = 20,395 (96.3% of the subsample) in depression model. Analyses were also run with an additional category for missing values for all variables to test whether results were changed. Theses analyses yielded similar results to those presented in the study. We also performed sensitivity analyses excluding the three countries in which the sampling frame was different (Austria, Greece, and Switzerland). The results were again similar to those presented below. A previous study reported that attributing a survival effect in an observational study should be considered as a tentative [25]. Therefore, we confirmed our results using mixed-effect logistic regressions, with the health outcome considered as a binary variable (e.g., death/not death). The results were similar, so we could be confident in the results using survival times. Analyses were performed using R version 3.4.0 and the package “coxme” version 2.2–5 for mixed-effects Cox models.

## Results

In the complete cohort used to assess the risk of mortality, a total of 14.6% of the participants (n = 4,257) died during the study at, on average, 79.1 years old ± 10.4 years. In the cohort without physical limitations at baseline, 37.5% of the participants (n = 6,332) developed global activity limitations at, on average, 69.2 years old ± 9.5 years. In cohort without depression at baseline, 22.4% of the participants (n = 4,742) were diagnosed with depression by the end of the study, with a mean age of diagnostic at 70.1 years old ± 10.0 years.

At baseline, more than half of the participants did not have a voluntary private insurance to cover expenses not covered by the country’s health insurance (52.1% of the total sample), and 62.3% reported out-of-pockets expenditures (30.9% reported having paid 200€ or more). Only 4.8% of the participants of the total sample reported to forgo health care for economic reasons. Other descriptive statistics are reported in Table 2. S1 Table reports the associations of socio-demographic, health, and health care-related variables with voluntary private insurance.

**Table 2:** Baseline characteristics of the study samples (individual level).

	Cohort		
	Complete	Physical health	Mental health
	n = 29260	n = 16879	n = 21178
**Socio-demographic variables**			
Country			
Austria	5.2 (1528)	4.6 (783)	5.7 (1204)
Belgium	12.4 (3640)	13.2 (2222)	12.8 (2712)
Denmark	5.5 (1614)	5.1 (867)	6.1 (1300)
France	10.1 (2965)	10.5 (1767)	8.7 (1846)
Germany	10.0 (2930)	8.7 (1475)	11.1 (2343)
Greece	9.1 (2667)	11.0 (1858)	9.1 (1921)
Israel	7.9 (2306)	8.0 (1350)	6.8 (1442)
Italy	8.6 (2506)	8.9 (1497)	7.7 (1640)
Netherland	9.8 (2872)	9.3 (1571)	10.6 (2245)
Spain	7.8 (2284)	7.1 (1198)	6.5 (1382)
Sweden	10.2 (2996)	9.9 (1665)	11.2 (2376)
Switzerland	3.3 (952)	3.7 (626)	3.6 (767)
Age at entry, mean (standard deviation)	64.6 (10.1)	62.5 (9.1)	63.9 (9.6)
Sex			
Female	54.2 (15854)	51.2 (8646)	49.1 (10401)
Male	45.8 (13406)	48.8 (8233)	50.9 (10777)
Marital status			
Single/divorce/widow	27.9 (8162)	24.5 (4141)	24.3 (5148)
Married/couple	71.8 (21013)	75.5 (12735)	75.7 (16028)
Did not answer	0.3 (85)	<0.01 (3)	<0.01 (2)
Income			
First quartile	23.0 (6738)	20.8 (3516)	20.3 (4305)
Second quartile	23.0 (6739)	21.8 (3682)	22.7 (4801)
Third quartile	23.0 (6739)	23.9 (4034)	24.7 (5240)
Fourth quartile	23.0 (6738)	25.5 (4297)	25.5 (5390)
Did not answer	8.0 (2306)	8.0 (1350)	6.8 (1442)
**Health variables**			
Depression			
No	72.4 (21178)	83.1 (14032)	100 (21178)
Yes	24.9 (7283)	15.5 (2617)	-
Did not answer	2.7 (799)	1.4 (230)	-
Physical limitations			
No	57.7 (16879)	100 (16879)	66.3 (14032)
Yes	41.9 (12246)	-	33.7 (7145)
Did not answer	0.5 (135)	-	<0.01 (1)
Lifetime daily smoking			
No	52.8 (15379)	51.7 (8717)	51.0 (10795)
Yes	47.2 (13733)	48.3 (8156)	49.0 (10382)
Sport			
No	41.8 (12154)	30.7 (5183)	36.0 (7611)
Yes	58.2 (16949)	69.3 (11682)	64.0 (13561)
**Health care-related variables**			
Forgone care because of cost			
No	94.5 (27651)	96.6 (16299)	97.1 (20561)
Yes	4.8 (1397)	3.2 (540)	2.9 (607)
Did not answer	0.7 (212)	0.2 (40)	<0.01 (10)
Having a voluntary private insurance			
No	52.1 (15243)	48.8 (8174)	51.4 (10895)
Yes	44.1 (12910)	47.8 (8064)	44.9 (9511)
Did not answer	3.8 (1107)	3.8 (641)	3.6 (772)
Having out-of-pocket expenditures			
No	27.3 (8002)	31.0 (5224)	27.9 (5898)
1–199€	56.3 (16460)	56.1 (9466)	58.1 (12297)
≥ 200€	6.1 (1788)	4.2 (718)	5.0 (1067)
Did not answer	10.3 (3010)	8.7 (1471)	9.0 (1916)

Percentage and n under brackets are reported, unless otherwise specified.

Baseline data: 2004.

Regarding system-level factors (Table 1), five out of twelve countries had an insurance mandate system, four a two-tier system, and three a single-payer system. The types of voluntary private insurances were very different between countries, and even between countries with a same health insurance system.

Individual-level factors were associated with risk of mortality and risks for physical limitations and depression (Table 3). Controlling for other individual factors, having a voluntary private insurance was protective (0.87≤HR≤0.91), whereas having out-of-pocket expenditures was a risk factor (1.14≤HR≤1.21 when comparing the reference category 0€ with the category expenses ≥ 200€, result marginally significant for risk of depression). However, having no expenses was a risk factor of mortality compared to having expenses between 1 and 199€ (HR = 1.12). It was a protective factor for physical limitations (HR = 0.89) and there was no significant relationship for depression (HR = 0.94). Since we controlled for demographics and socioeconomic factors (income, gender, age, marital status, and health), health care factors appeared as independently associated with risks of mortality, physical limitations, and depression.

**Table 3:** Hazard ratios for associations of individual and system-level factors with mortality, physical limitations, and depression, during the follow-up period 2004–2015 in twelve countries.

	Mortality		Physical limitations		Depression	
	Hazard ratio	p-value	Hazard ratio	p-value	Hazard ratio	p-value
**System-level factors**						
Type of UHC (ref. single payer)						
Insurance mandate	-	-	-	-	-	-
Two-tier	-	-	-	-	-	-
**Individual-level factors: health care factors**						
Voluntary private insurance (ref. no)	0.91	.037	0.90	.002	0.87	< .001
Out-of-pocket expenditures (ref. 1–199€)						
0€	1.12	.023	0.89	.005	0.94	.096
≥ 200€	1.21	< .001	1.15	< .001	1.14	.054
Did not answer	0.83	.035	1.01	.910	1.02	.720
Forgone care because of cost (ref. no)	0.83	.020			1.21	.013
**Individual-level factors: socio-demographics**						
Age	1.09	< .001	1.03	< .001	1.01	< .001
Gender (ref. female)	1.61	< .001	-	-	0.66	< .001
Marital status (ref. single)	0.88	< .001	-	-	-	-
Income (ref. first quartile)						
Second quartile	0.89	.008	0.91	.016	0.97	.460
Third quartile	0.81	< .001	089	.003	0.92	.068
Fourth quartile	0.83	.002	0.86	< .001	0.87	.004
Did not answer	1.18	.650	0.73	.370	1.51	.820
**Individual-level factors: health factors**						
Global activity limitation (ref. no)	1.47	< .001	-	-	1.51	< .001
Depression (ref. no)	1.28	< .001	1.36	< .001	-	-
Lifetime smoking	1.29	< .001	-	-	-	-
Sport (ref. never/hardly never)	0.70	< .001	0.93	.006	0.87	< .001

Mixed-effects Cox proportional hazards models (Efron method) with backward selection were performed. Only significant variables are included in the final models.

Age was associated with an increased risk of mortality and risks for physical limitations, and depression (1.01≤HR≤1.09). Being male was a risk factor for mortality (HR = 1.61) and protective against risk of depression (HR = 0.66). There was no significant difference for physical limitations and this variable was not included in the final model. Being in a couple was protective against the risk of mortality (HR = 0.88), but there was no significant difference with single participants for physical limitations and depression, and this variable was excluded from these models. Participants with higher income were less at risk for all outcomes compared to participants with lower income (0.81≤HR≤ 0.91, difference only between the first and fourth quartile for the risk of depression). Having health problems was a risk factor for mortality (global activity limitation, HR = 1.47; depression, HR = 1.28) and for health outcomes (depression for physical limitations, HR = 1.05; physical limitation for depression, HR = 1.51). Lifetime smoking was a risk factor only for the risk of mortality (HR = 1.29). Sport was protective for all outcomes (0.70≤HR≤0.93). Forgoing care due to cost was also a risk factor for the risks of physical limitations and depression (HR = 1.21 and 1.22). On the contrary, it was a protective factor against the risk of mortality (HR = 0.83).

The system-level factors (type of health insurance and type of voluntary private insurance) were not related to health outcomes and these variables were removed from the final models.

## Discussion

The aim of this study was to investigate health inequalities among middle-aged and elderly people in a large, population-based cohort in countries with UHC. Health care-related factors were associated with health status, measured using mortality and indicators of physical and mental health (physical limitations and depression). The preliminary results showed that even in these countries, financial burden related to health care was likely to occur: 44.1% of the participants took a voluntary private health insurance to cover health expenses not included in the public or compulsory health care system and 62.4% reported out-of-pockets expenditures.

### Health status and voluntary private health insurance

At the individual level, voluntary private health insurance was consistently associated with survival times of health outcomes. Having a private health insurance was associated with decreased risk of mortality and decreased risks for physical limitations and depression. Since the model controlled for income and other socio-demographic factors, having a voluntary private insurance was not a proxy for socioeconomic level: people with a similar income having a voluntary private insurance were more likely to be in good health and have a longer life than people without voluntary private insurance. However, this effect was of small amplitude. The main explanation of this association is that there is a causal mechanism between health financing, health care use, and health status. Having a voluntary private insurance provides an extended heath care coverage, which is likely to lead to use of preventive care and treatments. For example, women having a voluntary private insurance are more likely to use preventive care services [11].

Some countries consider voluntary private insurance as a key element of the health coverage system [26], but evaluations of private health insurances contrast. Previous findings showed that private insurances increase the system costs and enhance individual responsibility [27]. However, it also faces non-negligible issues: It is likely to exclude low-income people from access to care, and to increase overall health-related expenditures. From the health inequalities perspective, voluntary private insurance might be a benefit for middle-aged and elderly people.

### Health status and out-of-pocket expenditures

Out-of-pocket expenditures also appeared to be a factor of health inequality, as having important expenditures was a risk for mortality, physical limitations, and depression, with again small effect sizes. Previous studies in countries with UHC reported that out-of-pocket expenditures are associated with the burden of illness [28] and that elderly people with multiple chronic conditions are likely to be unable to afford out-of-pocket expenses [29]. However, to our knowledge, no studies highlighted how out-of-pocket expenditures directly affect mortality and health outcomes among healthy middle-aged and elderly people while taking into account others well-known confounders such as income or health. Small out-of-pocket expenditures (< 200€) were not consistently associated with premature health problems. Indeed, it was protective against the risk of mortality. It is possible that this variable also captured whether people used preventive care.

### Health status and kind of UHC

Health care factors measured at the system level were not related to health status: single-payer, two-tier, and insurance mandate systems displayed were not different from one another. Previous studies reported multiple advantages of a single-payer system compared to a multi-payer system, such as efficiency in revenue collection, overall cost control, prevention of selection, and increase in national solidarity [30]. Multi-payer systems have fewer advantages, such as a greater diversity of insurance products, flexibility, competition between providers, and citizens’ empowerment [30]. These systems were comparable when focusing on health status among middle-aged and elderly people.

### Health status and others socio-demographic variables

Finally, this study replicated previous findings regarding associations between individual risk factors of health inequalities [8]: gender (males being more likely to die earlier than women), marital status (single participants being more likely to have a premature death than participants in couple), income (low-income participants being more likely to die prematurely than high-income participants), and illnesses (participants with physical limitations, depression, and at-risk health behaviors [smoking and no sport activity] being more likely to die earlier than participants without physical limitations, depression, and at-risk health behaviors). The risk factors were almost the same for physical limitations (except gender, marital status, and smoking) and depression (except marital status and smoking). Forgoing care due to cost showed inconsistent results. It was a risk factor for health outcomes, with a small effect size. This result was in line with previous findings. Cross-sectional studies reported that health care forgoing due to financial reasons is associated with worse health outcomes [10, 31–34]. It also showed that forgoing care due to cost was associated with a decreased risk of mortality. Forgoing care is probably not a protective factor for risk of mortality: it is possible that participants who forwent care were those with minor illnesses, whereas those who did not forgo care had major health problems, leading to a premature death. Future studies are needed to analyze type of care participants forgo to achieve a better understanding of this phenomenon.

### Limitations

Our study had some limitations. First, we were unable to identify undiagnosed global activity limitation and depression, which might have led to underestimation of the true effects. Additionally, we used the EURO-D—a tool designed to assess depression—was used as a proxy for mental health. Therefore, it excluded other mental health problems. Further studies should include a larger range of tools to assess mental health problems to provide a more extensive overview of its association with health status. In addition, it would help to address the unobserved heterogeneity, i.e., variations dues to omitted variables. Other variables related to physical health, lifestyles, and anticipation of health care needs should also be included to provide a better understanding of the associations of health care-related factors with health status. Another limitation was that all questions were self-reported ones, and participants may have under- or over-reported certain problems. Results should thus be interpreted with caution. Finally, the response rate was quite low for some countries (especially Switzerland and Belgium). However, we performed analyses excluding these countries, and the findings remained similar.

### Conclusion

This population-based cohort from a large range of countries confirmed that health inequalities remained, even in countries with UHC [4, 7], and showed that some of these inequalities are caused by health care factors. These findings suggest guidance to reform in health care systems and public health actions to reduce health inequalities from a health care perspective. Furthermore, middle-aged and elderly people should not be neglected in health inequality research [14], since they are a vulnerable stage of life for health burden. Health care variables should be at focus in future researches designed to address health inequalities.

## Supporting information

S1 Table Baseline comparisons between participants with and without voluntary private insurance. (DOCX)
