# Supplementary material for: Associations of lack of voluntary private insurance and out-of-pocket expenditures with health inequalities. Evidence from an international longitudinal survey in countries with universal health coverage
Source: PLoS One. 2018 Oct 9;13(10):e0204666. doi: 10.1371/journal.pone.0204666 (PMC6177131; doi:10.1371/journal.pone.0204666)
Supplement: S1 Table — (DOCX) [file pone.0204666.s001.docx]

Supplementary table 1. Baseline comparisons between participants with and without voluntary private insurance

|  |  | Voluntary private insurance | | p-value (effect size) |
| --- | --- | --- | --- | --- |
|  |  | No | Yes |  |
| **Socio-demographic variables** | |  |  |  |
| Age at entry, mean | | 65.5 | 63.6 | <.001 (.008) |
| Sex |  |  |  | .060 |
|  | Female | 54.7 | 45.3 |  |
|  | Male | 53.5 | 46.5 |  |
| Marital status | |  |  | <.001 (.023) |
|  | Single/divorce/widow | 56.0 | 44.0 |  |
|  | Married/couple | 53.4 | 46.6 |  |
| Income | |  |  | <.001 (.051) |
|  | First quartile | 57.1 | 42.9 |  |
|  | Second quartile | 54.6 | 45.4 |  |
|  | Third quartile | 55.4 | 44.6 |  |
|  | Fourth quartile | 50.3 | 49.7 |  |
|  | Did not answer | 51.4 | 48.6 |  |
| **Health variables** | |  |  |  |
| Depression | |  |  | <.001 (.028) |
|  | No | 53.4 | 46.6 |  |
|  | Yes | 56.6 | 43.4 |  |
| Physical limitations | |  |  | <.001 (.089) |
|  | No | 50.3 | 49.7 |  |
|  | Yes | 59.3 | 40.7 |  |
| Lifetime daily smoking | |  |  | <.001 (.027) |
|  | No | 55.4 | 44.6 |  |
|  | Yes | 52.7 | 47.3 |  |
| Sport | |  |  | <.001 (.063) |
|  | No | 57.8 | 42.2 |  |
|  | Yes | 51.5 | 48.5 |  |
| **Health care-related variables** | |  |  |  |
| Forgone care because of cost | |  |  | .028 (.013) |
|  | No | 54.3 | 45.7 |  |
|  | Yes | 51.3 | 48.7 |  |
| Having out-of-pocket expenditures | |  |  | <.001 (.060) |
|  | No | 50.6 | 49.4 |  |
|  | 1-199€ | 58.6 | 41.4 |  |
|  | ≥ 200€ | 55.6 | 44.4 |  |
|  | Did not answer | 44.9 | 55.1 |  |

Baseline data: 2004.

χ^2^ with Cramer’s V are reported, excepted for age (ANOVA and partial η^2^).
